# Immune development in late preterm neonates

**DOI:** 10.1186/1824-7288-40-S2-A40

**Published:** 2014-10-09

**Authors:** Gaetano Chirico

**Affiliations:** 1 Neonatology and Neonatal Intensive Care Unit, Children Hospital, Spedali Civili, Brescia, Italy

## 

Several recent studies have underlined that late preterm infants have a significantly increased risk of infection and sepsis, [1-3] mainly related to problems of adaptation from intra- to extra-uterine life of the immune defense mechanisms. Indeed, both the innate (natural, non specific) and the adaptive (acquired, specific) immune systems are incompletely developed at birth, the more preterm the neonate, the more severe and prolonged the immunodeficiency.[4,5]

T lymphocytes response to mitogens is poor, and T and B lymphocytes are immature: higher percentage of CD4^+^ T lymphocytes and lower of CD8^+^ cells, with a gradual decline with age of the CD4^+^/CD8^+^ ratio, and predominant naïve phenotype with elevated percentages of CD4^+^/CD45RA^+^ T cells; in addition, cytokine production is reduced and Th1-like response inadequate. The immaturity of lymphocytes and of antigen presenting cells are responsible for the marked deficiency of antibody production; also, levels of IgG are low in late preterm infants because transplacental passage from the mother mostly occurs during the last trimester of gestation; therefore, these neonates may lack the protection ensured by maternal derived pathogen-specific IgG. The inability to produce adequate amounts of hematopoietic growth factors, particularly G- and GM-CSF, and the reduced neutrophil, complement and natural killer cell activity, may further amplify the neonatal impairment of immune defenses.

The combined neonatal deficiency of immunoglobulin, complement and neutrophil activity results in increased susceptibility to systemic infections from encapsulated pathogens, such as Group B Streptococcus, Staphylococci and Klebsiella, that require opsonization for efficient phagocytosis and killing. The immaturity of pattern recognition receptors (PRR) response to pathogen-associated molecular patterns (PAMP), in particular the impaired TLR4 (Toll Like Receptor) signaling, [1] may contribute to the late preterm vulnerability to Gram-negative bacteria.[6]

It should be noted, however, that neonatal T cells are capable to raise type 1 and 2 immune responses upon appropriate stimulus. Neonatal immunization does not generally lead to rapid antibody responses, however, it may result in an efficient immunologic priming which can act as a basis for future responses. It is therefore possible to induce early protection by immunization at birth.[7]

Finally, to mitigate detrimental consequences of immunodeficiency in late preterm infants, it is of paramount importance to maintain the mother-newborn protective immunological link by ensuring the host of protective components provided by human milk.[8]
